# The relative contribution of diurnal and nocturnal pollinators to plant female fitness in a specialized nursery pollination system

**DOI:** 10.1093/aobpla/ply002

**Published:** 2018-01-17

**Authors:** Giovanni Scopece, Lucia Campese, Karl J Duffy, Salvatore Cozzolino

**Affiliations:** 1 Department of Biology, University of Naples Federico II, Naples, Italy; 2 Plant Conservation and Population Biology, Ecology, Evolution and Biodiversity Conservation Section, Leuven, Belgium

**Keywords:** *Hadena bicruris*, moth pollination, mutualism, nursery pollination, parasitism, pollination syndrome

## Abstract

Plants involved in specialized pollinator interactions, such as nursery pollination, may experience trade-offs in their female fitness, as the larvae of their pollinators may also consume seeds produced by the flowers they pollinate. These interactions could potentially shift between mutualism and parasitism, depending on the presence and abundance of both the nursery pollinator and of other pollinators. We investigated the fitness trade-off in a Mediterranean plant (*Silene latifolia*), which has a specialist nocturnal nursery pollinator moth (*Hadena bicruris*) and is also visited by several diurnal pollinators. We estimated the pollination rates and fecundity of *S. latifolia* in both natural and experimental populations in the Mediterranean. We estimated natural pollination rates in different flowering times and with presence/absence of the *H. bicruis* moth. Then by exposing plants to each pollinator group either during the day or at night, we quantified the contribution of other diurnal pollinators and the specialized nocturnal nursery pollinator to plant female fitness. We found no difference in plant fruit set mediated by diurnal versus nocturnal pollinators, indicating that non-specialist pollinators contribute to plant female fitness. However, in both natural and experimental populations, *H. bicruris* was the most efficient pollinator in terms of seeds produced per fruit. These results suggest that the female fitness costs generated by nursery pollination can be overcome through higher fertilization rates relative to predation rates, even in the presence of co-pollinators. Quantifying such interactions is important for our understanding of the selective pressures that promote highly specialized mutualisms, such as nursery pollination, in the Mediterranean region, a centre of diversification of the carnation family.

## Teaser

Nursery pollination is an unusual plant–insect interaction in which an insect is both pollinator and seed predator. Depending on the abundance of the nursery pollinator and of other pollinators, this interaction can range from mutualism to parasitism and it is thus likely to vary geographically. We investigated this mechanism in the widespread species *Silene latifolia* in a Mediterranean environment that is likely to offer a rich pollinator community to the plant, thus decreasing the dependence on the nursery pollinator. Surprisingly, we found that although generalist pollinators contribute significantly to plant fitness, the nursery pollinator is still the more efficient.

## Introduction

The availability and abundance of effective pollinators is a key factor in determining the fitness of animal-pollinated plants (Stephenson 1981; Muchhala 2003; Muchhala *et al.* 2009). Hence, it has been predicted that plants should evolve towards pollinator specialization by adapting to the more abundant and/or more effective pollinator species (Stebbins 1970; Crepet 1983; Schemske and Horvitz 1984). Despite this prediction, several studies that investigated pollinator-mediated selection on floral traits have demonstrated more complex and sometimes contradictory results (Morse and Fritz 1983; Jennersten 1988; Thompson and Pellmyr 1992; Groman and Pellmyr 1999; Herrera 2000; Young 2002; Wolff *et al.* 2003). This suggests that most specialized interactions are not a mere adaptation to the most effective pollinator or pollinator functional class (*sensu*Fenster *et al.* 2004) but selection may also favour floral traits that attract new pollinators without excluding previous ones (Waser *et al.* 1996; Aigner 2001, 2004; Fenster *et al.* 2004). This is the case, for instance, in plant species with both diurnal and nocturnal pollinators, which are adapted to main pollinators active in a specific time of the day, but also exploit co-pollinators acting in the alternative day period (Giménez-Benavides *et al.* 2007). In addition, pollinator availability in geographically widespread plants may vary among different environments (Herrera 1988; Fenster and Dudash 2001), thus producing locally divergent pollinator-mediated selection on already evolved flower traits (Bustamante *et al.* 2010). Consequently, the overall evolution of floral traits in a species can be the result of adaptation to many pollinators, even though individual populations at any moment in time would be continuously adapting to one or a few local pollinators (Dilley *et al.* 2000).

One of the more extraordinary examples of specialized plant–insect interactions is the nursery pollination system, where insects lay eggs and rear larvae in the fruit that resulted from their pollination (Thompson and Pellmyr 1992). This interaction, with varying degrees of specialization, has evolved independently in several plant lineages (Reynolds *et al.* 2012). In some cases, it is a strictly specialized system in which insect and plant traits are highly co-adapted (e.g. *Yucca* and *Tegeticula*, Phyllanthaceae and *Epicephala*, *Ficus* and Agaonidae wasps mutualisms; Pellmyr 2003; Kawakita 2010) to the extent that groups of plants and nursery pollinators involved in this interaction show parallel cladogenesis (Kawakita *et al.* 2004; Althoff *et al.* 2012; Cruaud *et al.* 2012). In other cases, plant–insect interactions are more general and reflect a less specialized stage of potential mutualism that can also shift to parasitism depending on the presence of effective co-pollinators (Dufay and Anstett 2003). This latter situation describes the interaction of night-pollinated *Silene latifolia* with its nursery pollinating *Hadena bicruris* moth (Brantjes 1976). These two species are widely distributed in Europe and occur in a range of different habitats (Bopp and Gottsberger 2004). However, most studies on *S. latifolia* have been conducted in non-native regions (mainly the USA) where *S. latifolia* has been introduced, but *H. bicruris* does not occur (Young 2002; Castillo *et al.* 2014), or in the northern part of its distributional range (Jürgens *et al.* 1996; Bopp and Gottsberger 2004; Dötterl *et al.* 2006; Burkhardt *et al.* 2012; Magalhaes and Bernasconi 2014). In these regions, the flowering time of *S. latifolia* closely matches with the period in which *H. bicruris* occurs in its adult phase. Indeed, previous work from north European population has shown that *S. latifolia* relies mainly on *H. bicruris* (Jürgens *et al.* 1996; Witt *et al.* 1999; Bopp and Gottsberger 2004; Dötterl *et al.* 2006).

In contrast, due to warmer climates in the Mediterranean, *S. latifolia* has a long flowering time spanning well beyond the emergence period of adult *H. bicruris* moths (Prieto-Benítez *et al.* 2017), hence there is a greater abundance of other insect taxa that may visit *S. latifolia* over its flowering season. Although *S. latifolia* has a typical moth pollination syndrome (e.g. pale flowers, scent emitted at night), diurnal flower visitors have been frequently recorded on flowers in the Mediterranean, which suggests that diurnal visitors may play a role in the pollination of *S. latifolia*. Indeed, given that it feeds on developing seeds of *S. latifolia*, the net effect of *H. bicruris* on the fitness of *S. latifolia* should be considered in the light of the pollination provided by other pollinators. As a consequence, the *S. latifolia*–*H. bicruris* interaction could potentially shift between mutualism and parasitism, depending also on variation in the services of secondary pollinators (Dufay and Anstett 2003; Giménez-Benavides *et al.* 2007).

We investigated pollination and reproductive success of *S. latifolia* in two Mediterranean populations, one with *H. bicruris* pollinators and one lacking them. Then we induced two pollination regimes at non-overlapping day–night periods in experimental plant populations in order to understand the relative importance of diurnal versus nocturnal pollinators. We compared their effects on plant reproductive success in terms of fruit and seed set. In this study, we specifically addressed the following questions: (i) Do natural populations of *S. latifolia* predated and non-predated by *H. bicruris* differ in seed set? (ii) Does the presence of *H. bicruris* enhance *S. latifolia* pollination efficiency? (iii) To what extent does *S. latifolia* depend on diurnal pollinators?

## Methods

### Study species

The white campion *S. latifolia* (= *Silene alba*) (Caryophyllaceae) is a short-lived perennial, dioecious plant native to Eurasia (Delph *et al.* 2002). Nocturnal insects (moths) and diurnal insects (e.g. hoverflies, bees, beetles) have been recorded as floral visitors. However, the specialist seed predating moth *H. bicruris* (Lepidoptera) is considered its main pollinator (Jürgens *et al.* 1996). Typically, female moths lay one egg per flower in female plants; once the larva emerges, it consumes the developing seeds within this primary fruit (i.e. the fruit of the flower in which the egg was laid). To complete development, one larva will consume 2–4 additional secondary fruits (i.e. fruits that the larvae move to and attack after leaving the primary fruit; Elzinga *et al.* 2005) on the same plant. Thus, only plants that have been primarily attacked experience secondary attacks. Moths discriminate between male and female plants and, as a rule, lay eggs in flowers on females (Brantjes 1976; Labouche and Bernasconi 2010).

### Natural populations

Between May and June 2011, we surveyed two natural populations located in Southern Italy (University Campus of Monte Sant’Angelo, Naples, hereafter referred to as MSA and Battipaglia, Salerno, hereafter referred to as BATT). For each randomly sampled individual, we recorded the number of seeds per fruit and the predation by *H. bicruris*. Predation was evaluated by recording the number of fruits showing presence of *H. bircuris* larvae. Fruits attacked by *H. bicruris* can be easily recognized either by the presence of a hole in the side of the fruit (a primary seed fruit) or by the presence of a large round hole on the top (a secondary seed fruit) (Elzinga *et al.* 2005). Those fruits directly predated by *H. bicruris* were excluded from seed set comparisons.

In a series of observations carried out during the month of May, we also collected and identified the most common diurnal floral visitors of *S. latifolia* in MSA. To do this, we randomly selected a patch of *S. latifolia* individuals and observed them for ~2 h at different times of the day between 8 and 19 h. Insects observed approaching flowers were captured with an insect net, killed and stored for subsequent identification. For all samples, the time of collection was recorded.

In 2013, in the MSA population, we estimated fruit and seed set of *S. latifolia* in terms of number of fruits produced and number of seeds per fruit during three consecutive months (sampling dates: 21 April, 15 May and 18 June) and recorded the rate of *H. bicruris* infestation over time. To do this, we quantified the total number of fruits produced and the number of fruits predated by *H. bicruris* in 21 (April), 19 (May) and 21 (June) plants, respectively. We then randomly selected five fruits on each sampled plant that showed no direct infection of *H. bicruris* and counted the seeds. In each month, we selected new flowering individuals in order to avoid re-collecting fruits persisting on plants, and to be sure that the collected fruits were produced by flower pollination in the sampling month.

### Pollinator exclusion experiment

In 2011, a total of 72 four-month-old *S. latifolia* plants were grown following Cozzolino *et al.* (2015) from seeds collected in the MSA population. Only female plants were used to test for the presence and effectiveness of diurnal and nocturnal pollinators by performing three treatments. To examine the effect of diurnal pollinators on female fitness, we covered 18 individuals with a small cage (2 mm mesh) every evening at dusk, and removed it the next morning at 8.00 a.m., repeating this process until all the flowers on those plants had completed flowering. Simultaneously, we performed the opposite experiment to test for nocturnal pollination: covering the nocturnal plants (17 individuals) every morning and removing cages in the evening. A set of 37 plants was used as a control treatment and was exposed to both diurnal and nocturnal pollinators. The rate of *H. bicruris* predation was measured as outlined above. All mature fruits were counted and collected from plants of the three treatments for subsequent seed counting.

### Statistical analyses

In the natural populations (MSA and BATT), we compared plants predated and non-predated by *H. bicruris* in terms of mean number of seeds per fruit. Further, in the MSA natural population, we compared fruit and seed set among three consecutive months. Finally, we compared fruit and seed set in experimental plant populations exposed to diurnal pollination, nocturnal pollination or open pollination. Mann–Whitney *U*-tests were used to determine if the differences in the medians of analysed groups were significant. For multiple comparisons, we used a Kruskal–Wallis test with Mann–Whitney *U*-tests for *a posteriori* pairwise comparison with the significance level set to 0.01 (Bonferroni correction). All analyses were carried out in SPSS ver. 22 (SPSS Inc., Chicago, IL, USA).

## Results

### Natural populations

**Figure 1.**
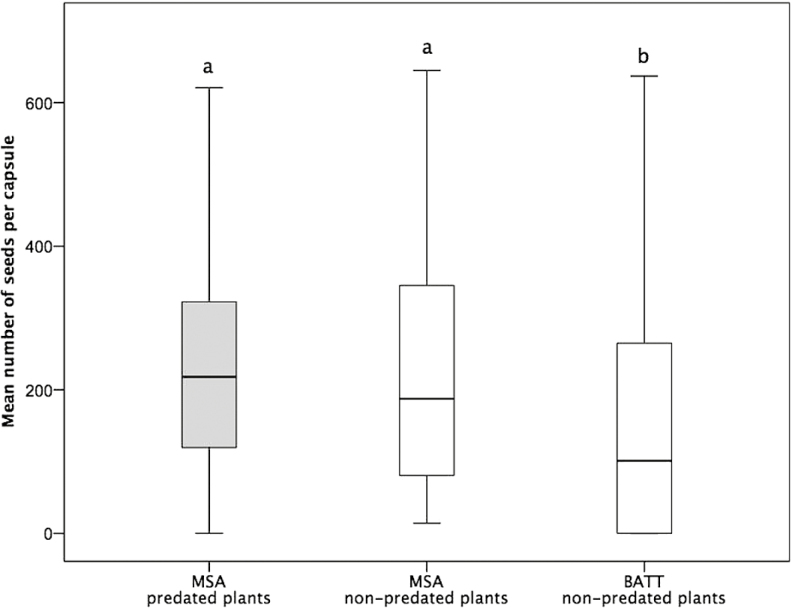
Mean number of seeds per fruit in *S. latifolia* plants predated (grey boxplots) or non-predated (white boxplots) by *H. bicruris* in MSA and BATT populations. Different letters indicate significant differences.

In MSA population, 14 out of 69 plants showed *H. bicruris* predation. All 21 individuals sampled in the BATT population were non-predated. We found no differences in number of seeds per fruit in predated and non-predated individuals in MSA (*U* = 2462.0; *Z* = −0.705; *P* = 0.481). Fruits from BATT contained significantly fewer seeds compared with both predated (*U* = 1669.5; *Z* = −3.190; *P* = 0.001) and non-predated (*U* = 7140.0; *Z* = −4.646; *P* < 0.001) plants in the MSA population (Fig. 1). Main diurnal floral visitors were Coleopteran (47.4 % mainly belonging to the genus *Oedemera*), Hymenopteran (42.1 % mainly belonging to the genus *Halictidae*) and more rarely Dipteran (10.5 %).

Fruit and seed set of *S. latifolia* varied over its flowering season (3 months) in the MSA population (Kruskal–Wallis test: fruit set χ^2^ = 9.224; DF = 2; *P* = 0.01; Kruskal–Wallis test: seed set χ^2^ = 50.806; DF = 2; *P* < 0.001). Number of fruits did not differ between April and May (*U* = 158.0; *Z* = −1.131; *P* = 0.270), but lowered in June (*U* = 139.0; *Z* = −2.073; *P* = 0.038 compared with April; *U* = 94.5; *Z* = −2.871; *P* = 0.004 compared with May).

**Figure 2.**
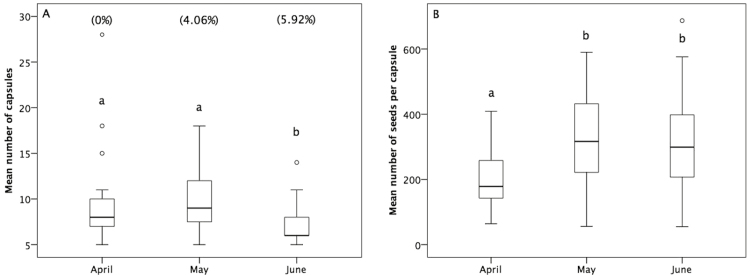
Comparison of fruit and seed set of *S. latifolia* plants in MSA population in three consecutive months. (A) Mean number of fruits per plant (in parenthesis the rate of fruits predated by *H. bicruris*). (B) Mean number of seeds per fruit. Different letters indicate significant differences. Circles represent outliers.

In April, mean number of seeds per fruit was significantly lower compared with May (*U* = 2230.0; *Z* = −6.161; *P* < 0.001) and June (*U* = 2431.5; *Z* = −6.286; *P* < 0.001). Mean number of seeds per fruit in May and June was not significantly different (*U* = 5234.5; *Z* = −0.493; *P* = 0.622) (Fig. 2). There was no predation of plants by *H. bicruris* in April and equal levels of plant predation in May (8 out of 197 fruits; 4.06 %) and June (9 out of 152 fruits; 5.92 %).

### Pollinator exclusion experiment

Ten out of 18 plants (58.8 %) exposed to nocturnal pollinators and 8 out of 37 open-pollinated plants (21.6 %) were predated by *H. bicruris*. The diurnal-only plant treatment experienced no *Hadena* infection.

**Figure 3.**
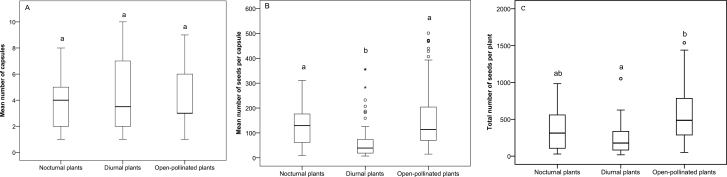
Comparison of fruit and seed set in experimental populations of *S. latifolia* with three pollination treatments: diurnal, nocturnal and open pollination. (A) Mean number of fruits per plant, (B) mean number of seeds per fruit and (C) total number of seeds per plant. Different letters indicate significant differences. Circles represent outliers.

Similar fruit set values were found in plants in the diurnal, nocturnal and open-pollinated treatments (Kruskal–Wallis test: χ^2^ = 0.06; DF = 2; *P* = 0.997; Fig. 3A). Number of seeds per fruit varied among treatments (Kruskal–Wallis test: χ^2^ = 54.597; DF = 2; *P* < 0.001; Fig. 3B): diurnal plant set produced the lowest amount of seeds per fruits when compared to nocturnal plant set (*U* = 714.5; *Z* = −4.546; *P* < 0.001) and open-pollinated plant set (*U* = 1970.0; *Z* = −7.323; *P* < 0.001); nocturnal plant set and open-pollinated plant set showed similar amount of seeds per fruit (*U* = 2627.5; *Z* = −0.395; *P* = 0.693).

The total number of seeds per plant marginally differed between the three treatments (Kruskal–Wallis test: χ^2^ = 10.51; DF = 2; *P* = 0.05; Fig. 3C): nocturnal and diurnal plant sets showed similar number of seeds per plant (*U* = 112.5; *Z* = −1.337; *P* = 0.184); number of seeds per plant in open-pollinated plant set did not differ from nocturnal plant set (*U* = 223.5; *Z* = −1.695; *P* = 0.09), but differed from diurnal plant set (*U* = 159.0; *Z* = −3.121; *P* = 0.002).

## Discussion

The *Silene*–*Hadena* interaction may range from mutualism to parasitism primarily depending on the plant fitness trade-off, although this also depends on the local abundance and efficiency of co-pollinators (Dufay and Anstett 2003). These factors may potentially offer the opportunity for *S. latifolia* to avoid *Hadena* pollination (and related seed predation) and increase the importance of a positive balance between seed predation and pollinator service for the evolutionary stability of such nursery interactions (Dufay *et al.* 2010).

In this study, we investigated the contribution of nocturnal and diurnal pollinators to *S. latifolia* pollination in the Mediterranean. We found that *S. latifolia* has similar levels of female reproductive success, even when its primary nocturnal pollinator was excluded from plants. However, plants exposed to nocturnal pollinators (i.e. including *H. bicruris*) produced more seeds per capsule, thus suggesting a higher efficiency of night pollinators. Overall, our results suggest that *S. latifolia* in the Mediterranean is less dependent on *H. bicruris* than has been seen in northern Europe. However, *H. bicruris* is still the most efficient pollinator, thus partially compensating for their predation costs even in the presence of ‘non-predator’ co-pollinators.

Mediterranean populations of *S. latifolia* may vary in the frequency and occurrence of *H. bicruris* as length of flowering season and availability of pollinators differ from northern European populations where most previous studies have been performed. By comparing two natural populations (MSA and BATT) that differed in the presence of *H. bicruris*, we found that the predated population of MSA produced more seeds per fruits than non-predated BATT population, indicating a higher pollination efficiency in the former population. Further, in MSA population, predated plants have a comparable number of seeds to non-predated plants suggesting that the pollination advantage was not exclusive of predated individuals. Since we compared only two natural populations (MSA and BATT), we cannot rule out other potential explanations for our findings including the presence of more pollinators or more co-pollinators or local differences in resource limitation.

In the Mediterranean, *S. latifolia* typically flowers almost all year, with a blooming peak from April to July, only partly overlapping with the flight period of adult *H. bicruris*. To test whether pollination success (in terms of seed and fruit number) was related to *H. bicruris* presence in the predated population of MSA, we estimated fruit and seed set and the occurrence of *H. bicruris*, in three consecutive months corresponding to the main blooming peak of *S. latifolia*. We detected the presence of *H. bicruris* both in May and June (as expected according with its emergence time in the Mediterranean) but not in April. Nevertheless, fruit set did not differ between April and May and decreased in June, indicating that in April pollinators other than *H. bicruris* actively contributed to *Silene* pollination. However, the number of seeds per fruit (a proxy of pollinator efficiency) was significantly higher in May and June than in April, matching the occurrence of *H. bicruris* in the MSA population. Taken together, these results suggest that predated and non-predated natural populations have comparable fruit set. Based on our results, we infer that this is due to contribution of other pollinators. However, *S. latifolia* has higher seed set when *H. bicruris* is present. This is in agreement with previous studies that found that moth visitors transfer pollen to stigmas more effectively than diurnal visitors (Young 2002; Reynolds *et al.* 2009).

The nocturnal/diurnal exclusion experiment demonstrated that both diurnal and nocturnal pollinators may potentially perform similar pollinator service in terms of seed set. However, the two pollination regimes differ in terms of fruit set and seed contents. Diurnally pollinated *S. latifolia* plants produce a comparable number of fruits to nocturnal and open-pollinated plants, suggesting that number of visitors/visits did not vary between treatments. However, night-pollinated *S. latifolia* plants (partly predated by *H. bicruris*) had a higher number of seeds per fruit than diurnal plants, thus suggesting a higher pollination efficiency of night pollinators (as *H. bicruris*). There were no significant differences between night-pollinated plants and open-pollinated plants; the latter plants benefitted from the pollination by both diurnal and nocturnal pollinators. This finding suggests a strong contribution of night pollination to the female fitness of the open-pollinated plants. Interestingly, similar findings were found a related *Silene ciliate–Hadena consparcatoides* nursery pollination system (Giménez-Benavides *et al.* 2007), suggesting a general trade-off trend for this type of pollination mechanism. Results from experimental populations with manipulated pollination regimes correspond well with those from the MSA population where the highest number of seeds per fruit was found when *H. bicruris* moths were present (i.e. on May and June).

Diurnal pollination has been widely reported in *Silene* (Giménez-Benavides *et al.* 2007; Reynolds *et al.* 2009) and in *S. latifolia* in particular (Young 2002). In a previous study carried out on the same population of MSA, seed set differed significantly between open-pollinated and hand-pollinated flowers, indicating that open-pollinated plants are pollen-limited (Cozzolino *et al.* 2015). Thus, there should be a selective pressure to increase seed set by exploring alternative (even less efficient) pollinators, such as diurnal ones. This may become particularly relevant when *Silene* plants are already blooming but *Hadena* is not available yet, as typically occurs in the Mediterranean climate. Indeed, our results show that before the appearance of *Hadena* (MSA population in April), or in the absence of *Hadena* (BATT population), *Silene* plants still achieve good pollination success. Although we cannot rule out the presence of nocturnal pollinators other than *Hadena*, these high levels of pollination success are likely the result of the contribution of diurnal pollinators.

The ecological context in which species interact may impose different selection pressures than those that initially led to the evolution of particular floral traits (Giménez-Benavides *et al.* 2007). Comparable female fitness of *S. latifolia* due to the availability of diurnal pollinators, even in the absence of its specialized nursery pollinator, suggests that net selection may sometimes promote a shift to diurnal pollinators and the exclusion of the nocturnal pollinating seed predator. Indeed, in the Mediterranean, the flowering of *S. latifolia* is not synchronous with *H. bicruris* emergence and abundance, but when available, *H. bicruris* does contribute to *S. latifolia* female fitness. We infer that the higher pollination efficiency of specialized pollinators may outweigh the potential contribution of short-term selective forces to avoid the nursery pollination, thereby allowing maintenance of *S. latifolia–H. bicruris* interaction in the Mediterranean. Although our study investigated cost and benefits of the pollinator relationships only for the female function, *Hadena* can also provide a benefit for male function, which is even more dependent on pollinator efficiency (Waelti *et al.* 2009; Litto *et al.* 2015). Potential conflicting selection between male and female flower functions may also help to explain why *H. bicruris* pollination is maintained also in the presence of other pollinators (Prieto-Benítez *et al.* 2017).

Taken together, our data suggest that *S. latifolia* is not obligately dependent on its nursery pollinator in the Mediterranean. In the absence of *H. bicruris*, *S. latifolia* has comparable female fitness to that observed when *H. bicruris* is present. This is particularly relevant as the flowering time of the plants significantly extends beyond the emergence *H. bicruris*, and generalist diurnal pollinators may provide *S. latifolia* with reproductive assurance during periods when the nursery pollinator is rare or absent. Nevertheless, Mediterranean *S. latifolia* plants use other (diurnal) pollinators but without excluding its specialized nursery pollinator despite cost of predation. This is a different outcome than seen in other *Silene* species. For example, pollination by *S. alba* in northern Europe is exclusively by specialized nocturnal pollinators. *Silene noctuiflora* is night-pollinated in its European range, yet also uses diurnal pollinators in its introduced range in North America (Witt *et al.* 1999). Several studies have shown the conditions that promote selection for nocturnal moth pollination and/or the switch to diurnal pollination in Caryophyllaceae (Jennersten 1988; Jennersten and Morse 1991; Shykoff and Bucheli 1995; Young 2002; Barthelmess *et al.* 2006).

## Conclusions

By showing that nursery pollination may incur female fitness costs that can be overcome through higher pollination rates relative to predation rates, our results provide an extra dimension to our understanding of dynamics of specialized plant–pollinator interactions and the cost/benefit balance of nursery pollination. Specialized pollinators are not always available, and some populations may co-opt generalist pollinators when they are abundant. These results emphasize the need to conduct such investigations on populations representing the distributional and diversity centres of the species under study (e.g. the Mediterranean for *Silene*; Giménez-Benavides *et al.* 2007), where pollinator abundance and diversity is likely to be high.

## Sources of Funding

This research was carried out in the frame of Programme STAR, financially supported by UNINA and Compagnia di San Paolo.

## Contributions by the Authors

G.S. conceived and designed the experiments, performed the experiments and wrote the paper; L.C. analysed the data and prepared figures and tables; K.J.D. contributed to data analysis and reviewed drafts of the paper; S.C. conceived and designed the experiments and reviewed drafts of the paper.

## Conflict of Interest

None declared.
